# *catena*-Poly[[diaqua­bis­(2-ethyl-1*H*-imidazole-κ*N* ^3^)cadmium]-μ-sulfato-κ^2^ *O*:*O*′]

**DOI:** 10.1107/S1600536812017059

**Published:** 2012-04-21

**Authors:** Chua-Hua Yu

**Affiliations:** a Orderd Matter Science Research Center, Southeast University, Nanjing 211189, People’s Republic of China

## Abstract

In the title one-dimensional coordination polymer, [Cd(SO_4_)(C_5_H_8_N_2_)_2_(H_2_O)_2_]_*n*_, the Cd^II^ atom (site symmetry 2) is coordinated by two sulfate O atoms, two water mol­ecules and two 2-ethyl­imidazole ligands in a distorted *cis*-CdN_2_O_4_ octa­hedral geometry. The water mol­ecules have a *cis* disposition. The bridging sulfate ions (site symmetry 2) link the Cd^II^ ions into a polymeric chain extending along [001]. The chains are linked by N—H⋯O and O—H⋯O hydrogen bonds. The terminal –CH_3_ group of the ligand is disordered over two orientations in a 0.61 (5):0.39 (5) ratio.

## Related literature

For background to ferroelectric materials, see: Zhang *et al.* (2010 ▶). For a related structure, see: Zhu & Yu (2011 ▶).
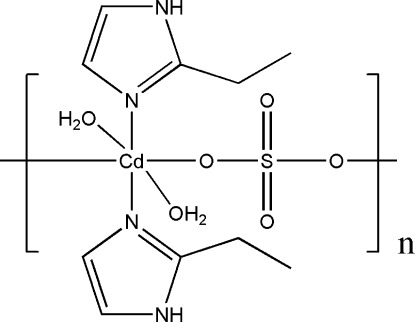


## Experimental

### 

#### Crystal data

[Cd(SO_4_)(C_5_H_8_N_2_)_2_(H_2_O)_2_]
*M* *_r_* = 436.78
Orthorhombic, 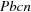

*a* = 14.465 (10) Å
*b* = 15.83 (1) Å
*c* = 6.990 (5) Å
*V* = 1600.6 (19) Å^3^
*Z* = 4
Mo *K*α radiation
μ = 1.53 mm^−1^
*T* = 293 K
0.34 × 0.28 × 0.24 mm

#### Data collection

Rigaku SCXmini diffractometer
Absorption correction: multi-scan (*CrystalClear*; Rigaku, 2005 ▶) *T*_min_ = 0.604, *T*_max_ = 0.693
15289 measured reflections
1838 independent reflections
1617 reflections with *I* > 2σ(*I*)
*R*_int_ = 0.035

#### Refinement

*R*[*F* ^2^ > 2σ(*F* ^2^)] = 0.035
*wR*(*F* ^2^) = 0.082
*S* = 1.06
1838 reflections
107 parameters
3 restraints
H atoms treated by a mixture of independent and constrained refinement
Δρ_max_ = 0.91 e Å^−3^
Δρ_min_ = −1.51 e Å^−3^

### 

Data collection: *CrystalClear* (Rigaku, 2005 ▶); cell refinement: *CrystalClear*; data reduction: *CrystalClear*; program(s) used to solve structure: *SHELXS97* (Sheldrick, 2008 ▶); program(s) used to refine structure: *SHELXL97* (Sheldrick, 2008 ▶); molecular graphics: *SHELXTL* (Sheldrick, 2008 ▶); software used to prepare material for publication: *SHELXL97*.

## Supplementary Material

Crystal structure: contains datablock(s) I, global. DOI: 10.1107/S1600536812017059/hb6691sup1.cif

Structure factors: contains datablock(s) I. DOI: 10.1107/S1600536812017059/hb6691Isup2.hkl

Additional supplementary materials:  crystallographic information; 3D view; checkCIF report

## Figures and Tables

**Table 1:** Selected bond lengths (Å)

Cd1—N2	2.255 (3)
Cd1—O2	2.437 (3)
Cd1—O1*W*	2.339 (3)

**Table 2:** Hydrogen-bond geometry (Å, °)

*D*—H⋯*A*	*D*—H	H⋯*A*	*D*⋯*A*	*D*—H⋯*A*
N1—H1*D*⋯O3^i^	0.86	2.11	2.936 (4)	160
O1*W*—H1*WA*⋯O3^ii^	0.81 (2)	1.93 (2)	2.732 (4)	172 (5)
O1*W*—H1*WB*⋯O3^iii^	0.80 (2)	1.97 (2)	2.762 (4)	169 (5)

Symmetry codes: (i) 
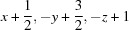
; (ii) 
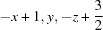
; (iii) 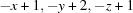.

**Fig. 1.**
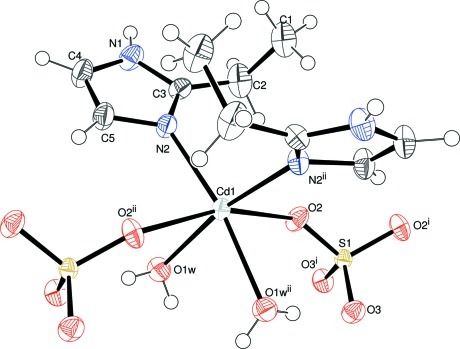
The molecular structure of the title compound, showing displacement ellipsoids drawn at the 50% probability level. Symmetry codes: (i) 1-x, y, 1/2-z; (ii) 1-x, y, 3/2-z.

**Fig. 2.**
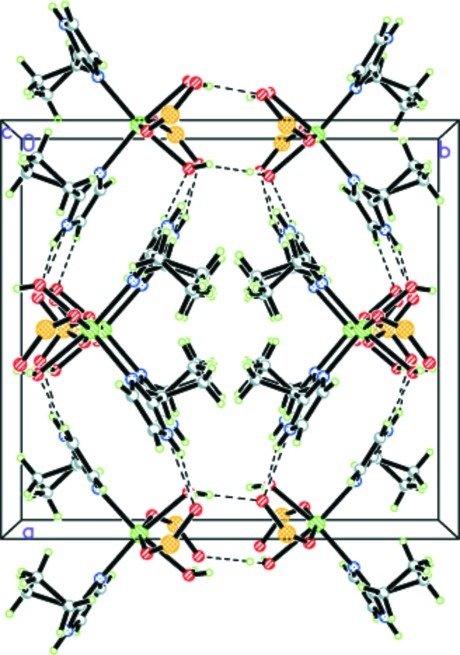
A view of the packing of the title compound, stacking along the c axis. Dashed lines indicate hydrogen bonds.

## supplementary crystallographic information

### Comment

According to finding potential ferroelectric phase change materials *via* dielectric constant measurements of compounds on the basis of temperature (Zhang *et al.*, 2010). Unluckily, no dielectric anomaly was observed ranging from 120 K to near 290 K. In this report the crystal structure of the title compound is herein reported. A structure chart showing the structure of the title compound (Scheme 1). A viewgraph of with the symmetry related fragments and atom-numbering scheme is shown in Fig. 1. The Cd(II) atom adopts two nitrogen atoms of two 2-ethylimidazole ligands [Cd1—N2 = 2.255 (3) Å], two O atoms from two water [Cd1—O1W = 2.339 (3) Å] and two oxygen atoms of two different sulfate radical ligands [Cd1—O2 = 2.437 (3) Å]. Besides, atom C1 swings between the two positions 1:1 ration. In addition, the unite exists O—H···O and N—H···O hydrogen bonds are also present (Table 1).

### Experimental

2.4 g (25 mmol) of 2-ethylimidazole was dissolved in 20 ml water, dropping 1.23 g (12.5 mmol) of H_2_SO_4_ into it, and 6.4 g (8.3 mmol) 3CdSO_4_.8H_2_O was added to the solution. After stirring the mixture for several minutes, 6.18 g (25 mmol) of Ba(NO_2_)_2_ was joined into it forming precipitation. The turbid liquid was filtered to give a light yellow solution. Yellow blocks were obtained, but not the expecting things, by the slow evaporation of the above solution after sever days at the ambient temperature.

### Refinement

All H atoms were placed in geometrically idealized positions and constrained to ride on their parent atoms but for H1A and H1B with C—H = 0.93 Å, with *U_iso_*(H) = 1.2 *U_iso_*(C).

### Crystal data

**Table:**

[Cd(SO_4_)(C_5_H_8_N_2_)_2_(H_2_O)_2_]	*F*(000) = 880
*M**_r_* = 436.78	*D*_x_ = 1.813 Mg m^−^^3^
Orthorhombic, *P**b**c**n*	Mo *K*α radiation, λ = 0.71073 Å
Hall symbol: -P 2n 2ab	Cell parameters from 1838 reflections
*a* = 14.465 (10) Å	θ = 3.9–27.5°
*b* = 15.83 (1) Å	µ = 1.53 mm^−^^1^
*c* = 6.990 (5) Å	*T* = 293 K
*V* = 1600.6 (19) Å^3^	Block, yellow
*Z* = 4	0.34 × 0.28 × 0.24 mm

### Data collection

**Table:**

Rigaku SCXmini diffractometer	1617 reflections with *I* > 2σ(*I*)
Radiation source: fine-focus sealed tube	*R*_int_ = 0.035
Graphite monochromator	θ_max_ = 27.5°, θ_min_ = 3.9°
ω scans	*h* = −18→18
Absorption correction: multi-scan (*CrystalClear*; Rigaku, 2005)	*k* = −20→20
*T*_min_ = 0.604, *T*_max_ = 0.693	*l* = −9→9
15289 measured reflections	3 standard reflections every 180 reflections
1838 independent reflections	intensity decay: none

### Refinement

**Table:**

Refinement on *F*^2^	Primary atom site location: structure-invariant direct methods
Least-squares matrix: full	Secondary atom site location: difference Fourier map
*R*[*F*^2^ > 2σ(*F*^2^)] = 0.035	Hydrogen site location: inferred from neighbouring sites
*wR*(*F*^2^) = 0.082	H atoms treated by a mixture of independent and constrained refinement
*S* = 1.06	*w* = 1/[σ^2^(*F*_o_^2^) + (0.0278*P*)^2^ + 5.5521*P*] where *P* = (*F*_o_^2^ + 2*F*_c_^2^)/3
1838 reflections	(Δ/σ)_max_ < 0.001
107 parameters	Δρ_max_ = 0.91 e Å^−^^3^
3 restraints	Δρ_min_ = −1.51 e Å^−^^3^

### Special details

**Table:**

Geometry. All e.s.d.'s (except the e.s.d. in the dihedral angle between two l.s. planes) are estimated using the full covariance matrix. The cell e.s.d.'s are taken into account individually in the estimation of e.s.d.'s in distances, angles and torsion angles; correlations between e.s.d.'s in cell parameters are only used when they are defined by crystal symmetry. An approximate (isotropic) treatment of cell e.s.d.'s is used for estimating e.s.d.'s involving l.s. planes.
Refinement. Refinement of *F*^2^ against ALL reflections. The weighted *R*-factor *wR* and goodness of fit *S* are based on *F*^2^, conventional *R*-factors *R* are based on *F*, with *F* set to zero for negative *F*^2^. The threshold expression of *F*^2^ > σ(*F*^2^) is used only for calculating *R*-factors(gt) *etc*. and is not relevant to the choice of reflections for refinement. *R*-factors based on *F*^2^ are statistically about twice as large as those based on *F*, and *R*- factors based on ALL data will be even larger.

### Fractional atomic coordinates and isotropic or equivalent isotropic displacement parameters (Å^2^)

**Table:**

	*x*	*y*	*z*	*U*_iso_*/*U*_eq_	Occ. (<1)
C1	0.5943 (16)	0.5775 (16)	0.464 (3)	0.0460 (9)	0.39 (5)
H1A	0.5329	0.6000	0.4539	0.069*	0.39 (5)
H1B	0.5961	0.5354	0.5625	0.069*	0.39 (5)
H1C	0.6117	0.5524	0.3440	0.069*	0.39 (5)
C1'	0.6100 (10)	0.5679 (10)	0.4805 (19)	0.0460 (9)	0.61 (5)
H1'A	0.6529	0.5216	0.4781	0.069*	0.61 (5)
H1'B	0.5778	0.5707	0.3607	0.069*	0.61 (5)
H1'C	0.5664	0.5596	0.5823	0.069*	0.61 (5)
C2	0.6611 (3)	0.6480 (3)	0.5124 (6)	0.0460 (9)	
H2A	0.7211	0.6343	0.4589	0.055*	
H2B	0.6399	0.6995	0.4513	0.055*	
C3	0.6724 (2)	0.6645 (2)	0.7199 (4)	0.0249 (7)	
C4	0.7286 (3)	0.6571 (2)	1.0116 (5)	0.0341 (8)	
H4	0.7662	0.6443	1.1155	0.041*	
C5	0.6496 (3)	0.7023 (2)	1.0139 (5)	0.0317 (8)	
H5	0.6230	0.7263	1.1220	0.038*	
N1	0.7422 (2)	0.63387 (19)	0.8256 (4)	0.0312 (6)	
H1D	0.7878	0.6045	0.7833	0.037*	
N2	0.61447 (19)	0.70720 (17)	0.8301 (4)	0.0250 (6)	
O1W	0.60396 (17)	0.90785 (16)	0.8035 (4)	0.0272 (5)	
O2	0.53001 (17)	0.82007 (15)	0.4109 (3)	0.0282 (5)	
O3	0.42204 (16)	0.92697 (15)	0.3118 (3)	0.0263 (5)	
S1	0.5000	0.87254 (6)	0.2500	0.0176 (2)	
Cd1	0.5000	0.797187 (19)	0.7500	0.02068 (12)	
H1WA	0.599 (3)	0.917 (3)	0.917 (3)	0.049 (14)*	
H1WB	0.594 (4)	0.9529 (17)	0.756 (6)	0.044 (14)*	

### Atomic displacement parameters (Å^2^)

**Table:**

	*U*^11^	*U*^22^	*U*^33^	*U*^12^	*U*^13^	*U*^23^
C1	0.039 (2)	0.061 (2)	0.0379 (18)	−0.0057 (17)	−0.0050 (15)	−0.0052 (16)
C1'	0.039 (2)	0.061 (2)	0.0379 (18)	−0.0057 (17)	−0.0050 (15)	−0.0052 (16)
C2	0.039 (2)	0.061 (2)	0.0379 (18)	−0.0057 (17)	−0.0050 (15)	−0.0052 (16)
C3	0.0262 (16)	0.0247 (15)	0.0240 (16)	0.0032 (12)	0.0015 (12)	0.0034 (12)
C4	0.0300 (19)	0.044 (2)	0.0285 (18)	0.0064 (15)	−0.0084 (14)	0.0021 (16)
C5	0.0338 (18)	0.0401 (19)	0.0214 (16)	0.0082 (15)	−0.0046 (14)	−0.0020 (14)
N1	0.0232 (14)	0.0384 (16)	0.0321 (15)	0.0094 (12)	0.0006 (12)	0.0029 (13)
N2	0.0249 (13)	0.0298 (14)	0.0202 (13)	0.0046 (11)	−0.0018 (11)	0.0004 (11)
O1W	0.0305 (12)	0.0280 (12)	0.0230 (12)	−0.0035 (8)	0.0025 (10)	0.0009 (10)
O2	0.0322 (12)	0.0342 (12)	0.0181 (11)	0.0069 (10)	0.0012 (9)	0.0066 (9)
O3	0.0244 (11)	0.0297 (12)	0.0248 (11)	0.0057 (9)	0.0018 (9)	0.0005 (9)
S1	0.0186 (5)	0.0204 (5)	0.0139 (4)	0.000	0.0004 (4)	0.000
Cd1	0.01904 (17)	0.02334 (18)	0.01966 (18)	0.000	−0.00215 (12)	0.000

### Geometric parameters (Å, º)

**Table:**

C1—C2	1.52 (2)	C5—N2	1.384 (4)
C1—H1A	0.9600	C5—H5	0.9300
C1—H1B	0.9600	N1—H1D	0.8600
C1—H1C	0.9600	O1W—H1WA	0.807 (19)
C1'—C2	1.485 (14)	O1W—H1WB	0.799 (19)
C1'—H1'A	0.9600	O2—S1	1.464 (2)
C1'—H1'B	0.9600	O3—S1	1.484 (2)
C1'—H1'C	0.9600	S1—O2^i^	1.464 (2)
C2—C3	1.483 (5)	S1—O3^i^	1.484 (2)
C2—H2A	0.9700	Cd1—N2	2.255 (3)
C2—H2B	0.9700	Cd1—O2	2.437 (3)
C3—N2	1.324 (4)	Cd1—O1W	2.339 (3)
C3—N1	1.341 (4)	Cd1—N2^ii^	2.255 (3)
C4—C5	1.348 (5)	Cd1—O1W^ii^	2.339 (3)
C4—N1	1.365 (5)	Cd1—O2^ii^	2.437 (3)
C4—H4	0.9300		
			
C2—C1—H1A	109.5	C3—N1—C4	108.6 (3)
C2—C1—H1B	109.5	C3—N1—H1D	125.7
H1A—C1—H1B	109.5	C4—N1—H1D	125.7
C2—C1—H1C	109.5	C3—N2—C5	106.2 (3)
H1A—C1—H1C	109.5	C3—N2—Cd1	130.0 (2)
H1B—C1—H1C	109.5	C5—N2—Cd1	122.4 (2)
C2—C1'—H1'A	109.5	Cd1—O1W—H1WA	104 (3)
C2—C1'—H1'B	109.5	Cd1—O1W—H1WB	119 (4)
H1'A—C1'—H1'B	109.5	H1WA—O1W—H1WB	103 (4)
C2—C1'—H1'C	109.5	S1—O2—Cd1	141.16 (14)
H1'A—C1'—H1'C	109.5	O2^i^—S1—O2	110.9 (2)
H1'B—C1'—H1'C	109.5	O2^i^—S1—O3	109.14 (14)
C3—C2—C1'	110.6 (6)	O2—S1—O3	109.34 (13)
C3—C2—C1	114.8 (9)	O2^i^—S1—O3^i^	109.34 (13)
C1'—C2—C1	11.3 (9)	O2—S1—O3^i^	109.14 (14)
C3—C2—H2A	108.6	O3—S1—O3^i^	109.0 (2)
C1'—C2—H2A	101.3	N2^ii^—Cd1—N2	101.65 (15)
C1—C2—H2A	108.6	N2^ii^—Cd1—O1W	170.07 (10)
C3—C2—H2B	108.6	N2—Cd1—O1W	87.78 (11)
C1'—C2—H2B	119.6	N2^ii^—Cd1—O1W^ii^	87.78 (11)
C1—C2—H2B	108.6	N2—Cd1—O1W^ii^	170.07 (10)
H2A—C2—H2B	107.5	O1W—Cd1—O1W^ii^	82.99 (14)
N2—C3—N1	109.9 (3)	N2^ii^—Cd1—O2^ii^	101.81 (9)
N2—C3—C2	126.1 (3)	N2—Cd1—O2^ii^	89.03 (10)
N1—C3—C2	123.9 (3)	O1W—Cd1—O2^ii^	81.24 (8)
C5—C4—N1	106.1 (3)	O1W^ii^—Cd1—O2^ii^	85.96 (9)
C5—C4—H4	127.0	N2^ii^—Cd1—O2	89.03 (10)
N1—C4—H4	127.0	N2—Cd1—O2	101.81 (9)
C4—C5—N2	109.3 (3)	O1W—Cd1—O2	85.96 (9)
C4—C5—H5	125.4	O1W^ii^—Cd1—O2	81.24 (8)
N2—C5—H5	125.4	O2^ii^—Cd1—O2	162.90 (12)

Symmetry codes: (i) −*x*+1, *y*, −*z*+1/2; (ii) −*x*+1, *y*, −*z*+3/2.

### Hydrogen-bond geometry (Å, º)

**Table:**

*D*—H···*A*	*D*—H	H···*A*	*D*···*A*	*D*—H···*A*
N1—H1*D*···O3^iii^	0.86	2.11	2.936 (4)	160
O1*W*—H1*WA*···O3^ii^	0.81 (2)	1.93 (2)	2.732 (4)	172 (5)
O1*W*—H1*WB*···O3^iv^	0.80 (2)	1.97 (2)	2.762 (4)	169 (5)

Symmetry codes: (ii) −*x*+1, *y*, −*z*+3/2; (iii) *x*+1/2, −*y*+3/2, −*z*+1; (iv) −*x*+1, −*y*+2, −*z*+1.
